# Circulating Exosomal microRNAs as Predictive Biomarkers of Neoadjuvant Chemotherapy Response in Breast Cancer

**DOI:** 10.3390/curroncol29020055

**Published:** 2022-01-28

**Authors:** Valentina K. Todorova, Stephanie D. Byrum, Allen J. Gies, Cade Haynie, Hunter Smith, Nathan S. Reyna, Issam Makhoul

**Affiliations:** 1 Division of Medical Oncology, University of Arkansas for Medical Sciences, Little Rock, AR 72205, USA; makhoulissam@uams.edu; 2 Department of Biochemistry and Molecular Biology, University of Arkansas for Medical Sciences, Little Rock, AR 72205, USA; sbyrum@uams.edu (S.D.B.); giesallenj@uams.edu (A.J.G.); 3 Biology Department, Ouachita Baptist University, Arkadelphia, AR 71998, USA; hay66232@obu.edu (C.H.); Smi66855@obu.edu (H.S.); reynan@obu.edu (N.S.R.)

**Keywords:** breast cancer, neoadjuvant chemotherapy, liquid biopsy, exosomal microRNA, predictive biomarkers, pathological complete response

## Abstract

Background: Neoadjuvant chemotherapy (NACT) is an increasingly used approach for treatment of breast cancer. The pathological complete response (pCR) is considered a good predictor of disease-specific survival. This study investigated whether circulating exosomal microRNAs could predict pCR in breast cancer patients treated with NACT. Method: Plasma samples of 20 breast cancer patients treated with NACT were collected prior to and after the first cycle. RNA sequencing was used to determine microRNA profiling. The Cancer Genome Atlas (TCGA) was used to explore the expression patterns and survivability of the candidate miRNAs, and their potential targets based on the expression levels and copy number variation (CNV) data. Results: Three miRNAs before that NACT (miR-30b, miR-328 and miR-423) predicted pCR in all of the analyzed samples. Upregulation of miR-127 correlated with pCR in triple-negative breast cancer (TNBC). After the first NACT dose, pCR was predicted by exo-miR-141, while miR-34a, exo-miR182, and exo-miR-183 predicted non-pCR. A significant correlation between the candidate miRNAs and the overall survival, subtype, and metastasis in breast cancer, suggesting their potential role as predictive biomarkers of pCR. Conclusions: If the miRNAs identified in this study are validated in a large cohort of patients, they might serve as predictive non-invasive liquid biopsy biomarkers for monitoring pCR to NACT in breast cancer.

## 1. Introduction

Breast cancer is the second leading cause of cancer death among women, with an annual increase rate of approximately 3% [1]. Neoadjuvant chemotherapy (NACT) is the systemic treatment of cancer prior to surgical therapy, designed to downstage the tumor. NACT for breast cancer is an established therapeutic option for selected high-risk, locally advanced breast cancers or to improve eligibility for breast-conserving surgery [2]. Approximately 25% of women diagnosed with localized breast cancer are subjected to NACT [3]. Currently used methods for therapy response cannot predict the treatment efficacy before the application of several therapy cycles, and a poor response could result in the unresectable tumor, metastatic tumor spread, and unnecessary toxicity [4]. Achievement of pathologic complete response (pCR) in breast cancer patients receiving NACT is associated with both overall survival and disease-free survival [5]. The standard approach to determine pCR to NACT is based on post-surgical histopathology, which quantifies the presence or absence of residual invasive cancer on hematoxylin/eosin-stained breast and lymph nodes specimens in response to therapy based on pre-treatment core biopsy and post-treatment surgical specimens [6]. A major benefit in the long-term outcome from achieving a pCR was found in patients with aggressive breast cancer subtypes, such as triple-negative breast cancer (TNBC) and HER2-positive, and hormone-receptor-negative [7]. Several non-invasive imaging methods that can determine whether there is residual disease could spare patients with pCR from having surgery. Common imaging techniques, including ultrasound (US), palpation, mammography, magnetic resonance imaging (MRI), and positron emission tomography/computed tomography, are available for response monitoring during NACT [8]. However, several reports indicate that the accuracy of the imaging modalities is insufficient to determine pCR to NACT [9,10,11]. Liquid biopsy-based biomarkers have recently been suggested as low-cost and minimally invasive biomarkers in cancer diagnostics [12] and the response to therapy [13] that may support the traditional monitoring tools. Patients who achieve pCR after NACT have a better long-term outcome [7]. However, non-responders do not benefit from continuing the treatment [14]. Moreover, the side effects of NACT can increase morbidity, and some patients can be rendered unfit for surgery or have their surgery delayed. Therefore, the early predictions of patients’ response to NACT can facilitate an opportunity to modify ineffective treatments with more effective ones on an individual patient basis. The early recognition of a non-response facilitates an early change to a non-cross-resistant regimen, thereby minimizing toxicity [15,16] and optimizing the timing of the surgery [17]. The associations between the molecular profiles of breast tumors before the treatment and the response to chemotherapy have been examined in several studies [18,19]. Vendrell et al. [20] compared the baseline molecular profile of breast tumors between a group of patients whose tumors failed to respond to endocrine therapy and a group of patients who remained disease-free for five years identified genes that were associated with a lack of response or resistance to endocrine therapy. Other studies have demonstrated that on-treatment biomarkers may be superior to those measured before exposure to treatment [21]. Bownes et al. [22] examined biopsies taken at diagnosis, at 2 weeks during NACT, at mid-chemotherapy, and at resection and found that on-treatment biomarkers had greater predictive accuracy than established prognostic tests.

Micro RNAs (miRNAs) are a class of small (~22 nucleotides) non-coding RNAs (ncRNAs), which regulate gene expression at the post-transcriptional level [23]. miRNAs regulate the expression of various downstream gene targets, including oncogenes, tumor suppressor genes, and transcription factors, thus exerting an impact on cancer initiation and progression [24]. miRNAs are present in the circulation in a very stable form, encapsulated into the extracellular vesicles (EVs), such as exosomes, thus being resistant to Rnase digestions and making them excellent biomarker candidates [25,26]. Circulating exo-miRNAs have been suggested as specific and stable molecular biomarkers in cancer therapy [27,28,29]. Evidence has shown that exosomes are released from most eukaryotic cells and, through their cargo of nucleic acids, proteins, and lipids, mediate cell-to-cell communications [30]. Exosomal cargo is specific for the originating cells and mirrors their physiological state, which makes exosomes promising diagnostic biomarkers. Tumor-derived exosomes released in the body fluids can travel to distant sites and affect the biological activities of different cell types, such as proliferation, invasion and metastasis, and stimulation of angiogenesis [31]. Recent studies have shown that circulating exo-miRNAs represent the ideal non-invasive biomarkers in liquid biopsy for monitoring of the disease progression or treatment efficacy [32]. The potential use of miRNAs as prognostic and therapeutic biomarkers in breast cancer has been the main focus of experimental and clinical research [33,34,35], but there is still a lack of consistency between the reports [36], and despite the increasing number of reported potential miRNA biomarkers, their practical application is still unclear [37]. Therefore, because of their great importance in the diagnosis, prognosis, and prediction of therapeutic responses miRNAs need to be updated [37].

This study aimed to characterize the miRNA profiles of plasma exosomes of breast cancer patients treated with NACT and to identify those that have the potential to predict pCR prior to and/or after the first cycle of NACT.

## 2. Materials and Methods

### 2.1. Study Population

A total of 20 breast cancer patients treated with NACT for invasive ductal carcinoma (IDC) from 2012 to 2018 were enrolled at the Winthrop Rockefeller Cancer Institute, UAMS. This study was approved by the Institutional Review Board (IRB) of UAMS (Protocol #130212) and from IRB of the Central Veterans Healthcare system (CAVHS) (Protocol #1423976-2), where the samples were processed and stored. All participants signed an IRB-approved informed consent where they were informed for the use of their blood samples and medical records for research purposes. The inclusion criteria included early ER+/PR+/Her2- or triple-negative, stage I to III breast cancers within 18–99 years of age. Participants were ineligible if they were pregnant or breastfeeding and had no prior history of chemotherapy or radiotherapy. All patients were treated with a predefined protocol, which included a combination of Adriamycin (60 mg/m^2^) with Cyclophosphamide (600 mg/m^2^) in each cycle for 4 cycles every 2 weeks, followed by surgical removal of the tumor. At the time of treatment, blood samples were collected prior to, and 14 days after the first cycle of NACT, plasma was isolated and stored at −80 °C. pCR was determined on surgical specimens and was defined as having no residual invasive carcinoma in the breast and no tumor in the axillary lymph nodes. Patient characteristics were obtained via retrospective hospital-based chart review and included age at diagnosis, tumor size on imaging, clinical axillary lymphadenopathy (i.e., biopsy-proven), histologic grade, immunohistochemistry (IHC) for estrogen/progesterone expression, HER2 expression via IHC or fluorescent in situ hybridization (FISH), therapy completion (yes/no), and pathological determination of pCR status. Patients were included in the study only if there were plasma samples available at both prior to and after first cycle of NACT. Of the 20 patients enrolled in this study, six (n = 6) patients had pCR and plasma samples available at both time-points. A total of fourteen (n = 14) patients with non-pCR and plasma samples available at both time points were also.

### 2.2. Plasma Exosomal miRNA Isolation and Next-Generation Sequencing (NGS)

Blood samples (10 mL) were collected in EDTA collection tubes and plasma was isolated within next 2 h by centrifugation at 2000 g for 20 min. Plasma was stored at −80 °C until analysis. Total RNA, including miRNA was isolated from 1 mL frozen plasma samples using exoRNeasy Serum/Plasma Maxi Kit (Qiagen, Valencia, CA, USA) [38]. Briefly, pre-filtered plasma (0.8 μm syringe filter) was mixed 1:1 with 2x binding buffer (XBP) and added to the exoEasy membrane affinity column to bind the EVs to the membrane. After centrifugation, the flow-through was discarded, and wash buffer (XWP) was added to the column to wash off non-specifically retained material. After another centrifugation and discarding of the flow-through, the vesicles were lysed by adding QIAzol to the spin column, and the lysate was collected by centrifugation. The miRNeasy Serum/Plasma Spike-In Control (Qiagen, Valencia, CA, USA) was added. Following the addition of chloroform, thorough mixing, and centrifugation to separate organic and aqueous phases, the aqueous phase was recovered and mixed with ethanol. The sample-ethanol mixture was added to Rneasy MinElute spin column and centrifuged. The column was washed once with buffer RWT, and then twice with buffer RPE followed by elution of RNA with water. The purity and concentrations of total RNA of the plasma samples were measured with NanoDrop ND-1000.

NGS libraries were constructed using a QIAseq miRNA library. Briefly, 3′ and 5′ adapters were ligated to mature miRNAs. The ligated miRNAs were then reverse transcribed to cDNA using a reverse transcription (RT) primer with unique molecular indices (UMI). After library amplification, a cleanup of the miRNA library was performed using a streamlined magnetic bead-based method and quality control (QC). The library was then sequenced on Illumina NextSeq 500/550 equipment.

### 2.3. Data Analysis

Sequence data were converted to FASTQ files, analyzed using CLC Genomics Workbench (v.12.02) and UMIs were extracted. Reads were mapped to MiRNA database miRbase v22 and human genome GRCh38 versions 97. Differential expression analysis was performed via the Bioconductor Package DESeq2, including hierarchical clustering plus heatmap, principal component analysis, normalization based on median ratios of mean miRNA expression, and the Benjamini–Hochberg method to correct for false discovery rate (FDR). miRNAs with post-treatment versus pre-treatment with FDR < 0.05 and log fold change (FC) > 1.0 were considered significant.

### 2.4. Target Predictions

miRNAs transcriptome targets were identified by TargetScan (http://www.targetscan.org/vert_71/ (accessed on 30 August 2020), miRDB (http://www.mirdb.org/) (accessed on 31 August 2020) and mirDIP (https://ophid.utoronto.ca/mirDIP/) (accessed 10 September 2020). DIANA-mirPath [39] was used for the KEEG pathway analysis of miRNA signature. DIANA-miRPath is a miRNA pathway analysis webserver that utilizes predicted miRNA targets provided by the DIANA-microT-CDS algorithm. Ingenuity pathway analysis (IPA) were used to visualize potential interaction networks for the differentially expressed miRNAs with their targets.

### 2.5. cBioportal and UALCAN Databases Analysis

The altered expressions of the selected miRNAs in breast cancer and the relative overall survival of the breast cancer patients were analyzed on publicly accessible data in cBiportal “https://www.cbioportal.org/ (accessed on 31 August 2020), Breast Invasive carcinoma (TCGA, PanCancer Atlas)” and Breast cancer (METABRIC) datasets and UACLAN database [40]. The survivability based on the copy number variations (CNVs) of the selected miRNAs in breast cancers was analyzed using cBioportal.

## 3. Results

### 3.1. Demographic Characteristics of the Study Participants

A total of 40 plasma samples of 20 patients with early-stage breast cancer were analyzed in this study. Clinical patient and tumor characteristics at the time of BC diagnosis are shown in Table 1. All breast cancer cases were histologically confirmed as early-stage invasive ductal carcinoma (IDC) of the breast with tumor size ranging between 2 and 4 cm. A total of 14 patients had estrogen receptor (ER) progesterone receptor (PR) positive and human epidermal growth factor receptor 2 (HER2) negative (ER+/PR+/Her2)-breast cancer, and 6 patients were diagnosed with triple-negative breast cancer (TNBC), including 3 patients with pCR and 3 patients with non-pCR. One patient in the non-pCR group diagnosed with stage III TNBC died two years after diagnosis due to brain metastasis. A total of 11 patients (5 in the pCR group and 6 in the non-pCR group) are still alive more than 5 years, and 8 are still alive 3–4 years from the initiation of the treatment.

### 3.2. Differentially Expressed Exosomal miRNAs

**Figure 1:**
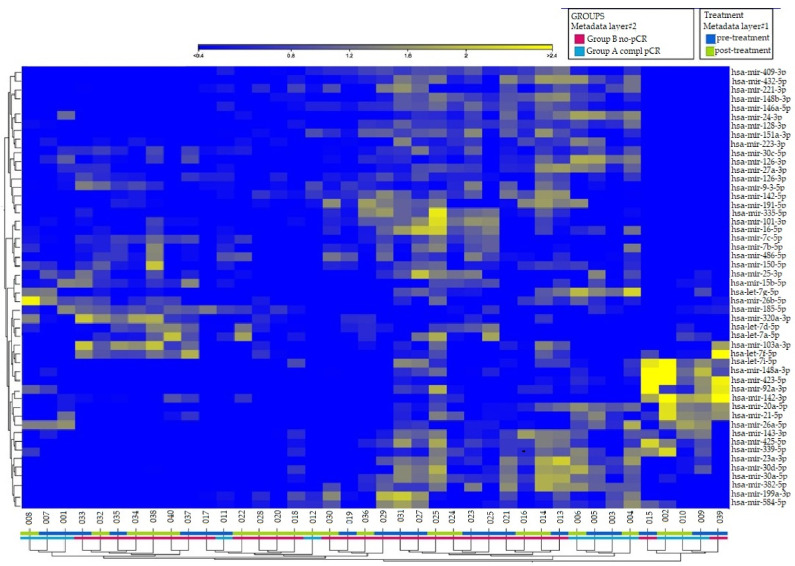
Hierarchical clustering on differentially expressed exo-miRNAs (*p*-value < 0.05, FDR < 2.0) in 40 samples collected prior to and post-first cycle from 20 breast cancer patients treated with NACT (doxorubicin/cyclophosphamide). The 50 miRNAs with the highest variance across samples were selected for unsupervised clustering. Each row represents one miRNA, and each column represents one sample. Patients’ clinical outcome and time of samples collection are indicated by colored squares at the bottom of the dendrogram: in layer#1 blue for pre-treatment (baseline) and green for post first cycle of NACT; in layer#2 red for the group of patients with non-pCR, and blue for patients with pCR. The intensity of the color is proportional to the degree of up- or downregulation. The more similar the expression of the selected genes are between samples, the closer the samples are related in the dendrogram.

Figure 1 shows the candidate miRNAs that discriminate between the groups of patients who achieved pCR and those who did not. In hierarchical clustering, genes with similar expression patterns are grouped together and are connected by a series of branches (clustering tree or dendrogram), and the length of the branches reflects the degree of similarity [41].

**Figure 2:**
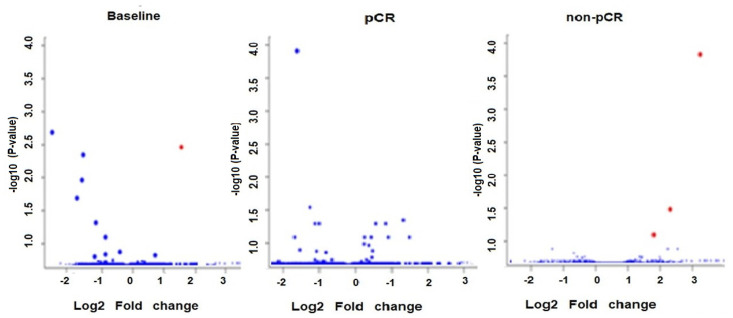
Volcano plot of circulating exo-miRs at baseline in pCR versus non-pCR and after the first NACT dose compared to baseline in the groups of patients with pCR and non-pCR. The plot is constructed by plotting the FDR corrected negative log10 (*p*-value) on the *y*-axis and the expression fold change between the two experimental groups on the *x*-axis. The larger the difference in expression of a feature, the more extreme its point will lie on the *x*-axis. The more significant the difference, the smaller the *p*-value and thus the higher the −log10 (*p*) value. Thus, points for features with highly significant differences will lie high in the plot. Features of interest are typically those which change significantly and by a certain magnitude. These are the points in the upper left and upper right-hand parts of the volcano plot. Red dots show upregulated miRNAs and blue dots show downregulated miRNAs.

The volcano plot in Figure 2 shows the relationship between the *p*-values of a statistical test and the magnitude of the difference in expression values of the samples in the groups. A total of eight circulating ex-miRNAs were predictive of the response to NACT in patients with early breast cancer. No significant differences were detected between the two groups of patients (pCR and non-pCR) with respect to the age, race, and breast cancer grade.

The analysis of differentially expressed exo-miRs at the baseline showed upregulation of mir-30b and downregulation of mir-423 and mir-328 in patients with pCR versus non-pCR (Table 2). Mir-127 was significantly upregulated in patients with TNBC who obtained pCR versus those who did not (Table 2). After the first NACT dose, the plasma of patients with pCR showed significantly downregulated mir-141 in comparison with the baseline, while the group with non-pCR showed upregulation of mir-34a and mir-183, and downregulation of mir-182 versus baseline (Table 2). A review of the literature presented in Supplemental Table S1 revealed dysregulation of these miRNAs in tumor tissue and/or circulation of breast cancer patients, as well as in breast cancer cell lines.

### 3.3. In Silico Target Prediction and Pathway Enrichment Analysis

**Figure 3:**
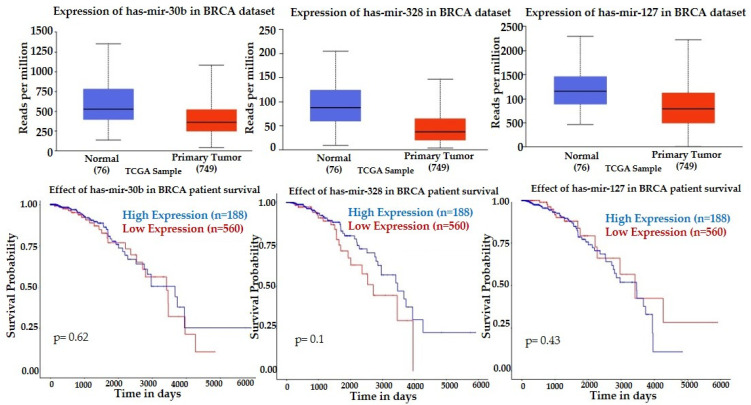
Expression profiles and prognostic value of mir-30, mir-328, and mir-127 in invasive breast cancer. Statistical significance of normal versus tumor 3.5649 × 10^−3^ (mir-30b), 7.3940 × 10^−10^ (mir-328) and 9.7639 × 10^−7^ (mir-127). Kaplan-Meier plots (UALCAN) show the effect of miRNAs expression in breast tumors and normal breast tissue on patients’ survival.

We have interrogated TCGA miRNA expression datasets for invasive breast cancer using UALCAN database. TCGA data indicate that invasive breast tumors have lower expression of mir-30b, mir-328, and mir-127 compared to normal breast tissue, and their lower tumor expression is associated with a better survival rate in the period of 6000 days (Figure 3).

**Figure 4:**
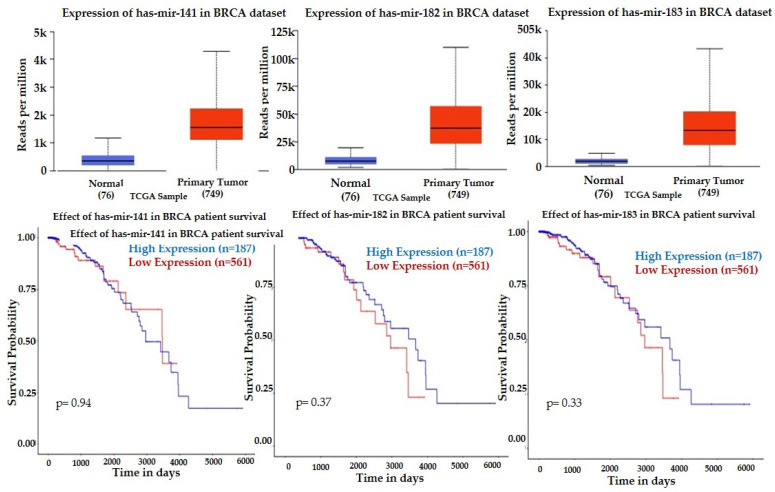
Expression profiles and prognostic value of mir-141, mir-182, mir-183, and in invasive breast cancer (BRCA) versus normal breast. Statistical significance of the expression of normal versus tumor 1.6244 × 10^−12^ for all three miRNAs. Kaplan–Meier plots (UALCAN) show the effect of high miRNAs expression and low miRNAs expression on a patient’s survival.

Lower expression of mir-30b and mir-328 were associated with better survival, which correlates with our findings, showing the trend of downregulation after the first NACT dose in patients with pCR (Table 2). UALCAN showed upregulation of mir-141, mir-182, and mir-183 in breast tumors compared to normal tissue, and their downregulation was associated with a higher survival rate (Figure 4), a finding that correlated with downregulation of mir-141 in patients with pCR at baseline (Table 2) and upregulation of mir-183 after the initial NACT in patients with non-pCR. However, the significant decrease of mir-182 after the initial NACT in the group of patients with non-pCR is not consistent with the published clinical data.

**Figure 5:**
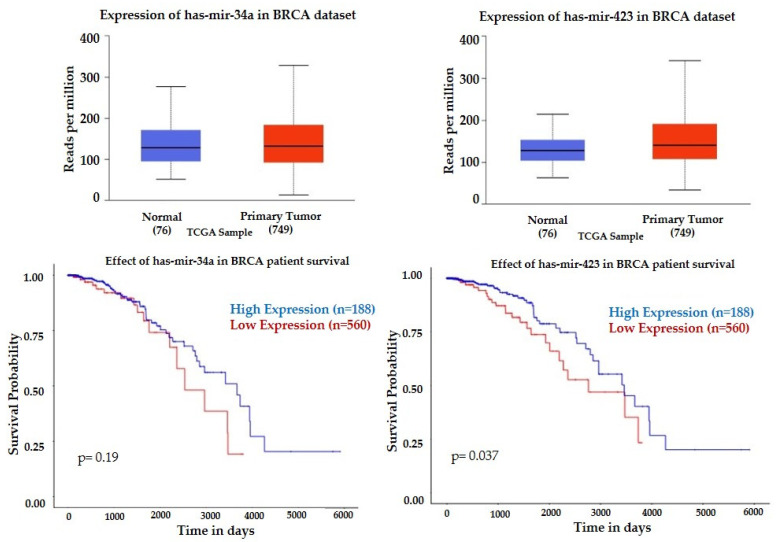
Expression profiles and prognostic value of mir-34a and mir-423 in breast tumors. Statistical significance of normal versus tumor 8.6303 × 10^−2^ (mir-34a) and 1.6551 × 10^−5^ (mir-423). Kaplan-Meier plots (UALCAN) show the effect of miRNAs expression on patients’ survival.

There is a similar expression of mir-34a and mir-423 between breast tumors and normal tissue, but downregulation of these miRNAs is associated with better survival prognosis (Figure 5), which correlate with the lower mir-423 at baseline pCR group with an insignificant elevation after the first NACT cycle, and higher mir-34a after the first NACT non-pCR group (Table 2). TCGA data indicate differing expression patterns for several miRNAs based on cancer subtypes, including lower expression of mir-183 and mir-141 in luminal breast cancer, lower 30b in HER2 positive, and lower mir-182, and mir-30b in TNBC compared to the other subtypes (Supplemental Figure S1). TCGA data also indicate a difference in the expression patterns for several miRNAs based on the nodal metastasis status (Supplemental Figure S2).

**Figure 6:**
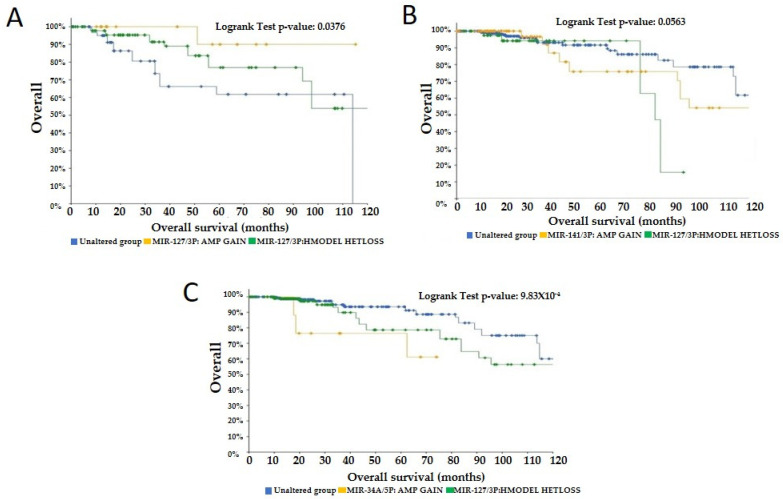
Kaplan–Meier plots (cBioPortal) showing the effects of CNV of mir-127 (**A**), mir-141 (**B**), and mir-34a (**C**) on breast cancer patients’ survival over 120 months.

To further explore the selected miRNAs, we used cBioPortal to determine the prognosis of breast cancer based on the expression levels and CNV data. cBioPortal’s Onco Query Language was used to integrate CNV amplification and gain cases into a Gain group, and CNV homozygous deletion and heterozygous loss cases into a Loss group. The analysis of the survival of breast cancer, based on CNV showed that CNV Gains of mir-127 were associated with better survival prognosis compared to CNV Loss, a finding which correlated with mir-127 significant upregulation in TNBC at baseline in our study (Figure 6A). CNV Loss in mir-141 (Figure 6B) and mir-34a (Figure 6C) were also associated with better survival than CNV Gains, correlating with mir-141 downregulation in pCR group and mir-34a upregulation in non-pCR. The rest of the candidate miRNAs did not show a clear correlation with the survival prognosis in correlation with the CNV gain and loss.

**Figure 7:**
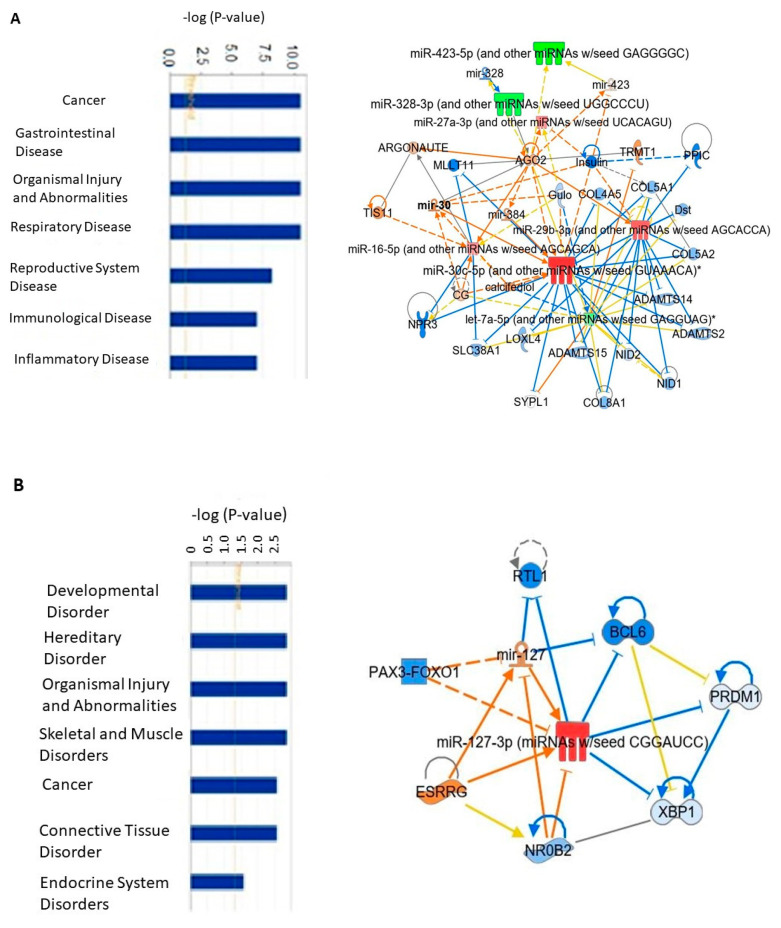

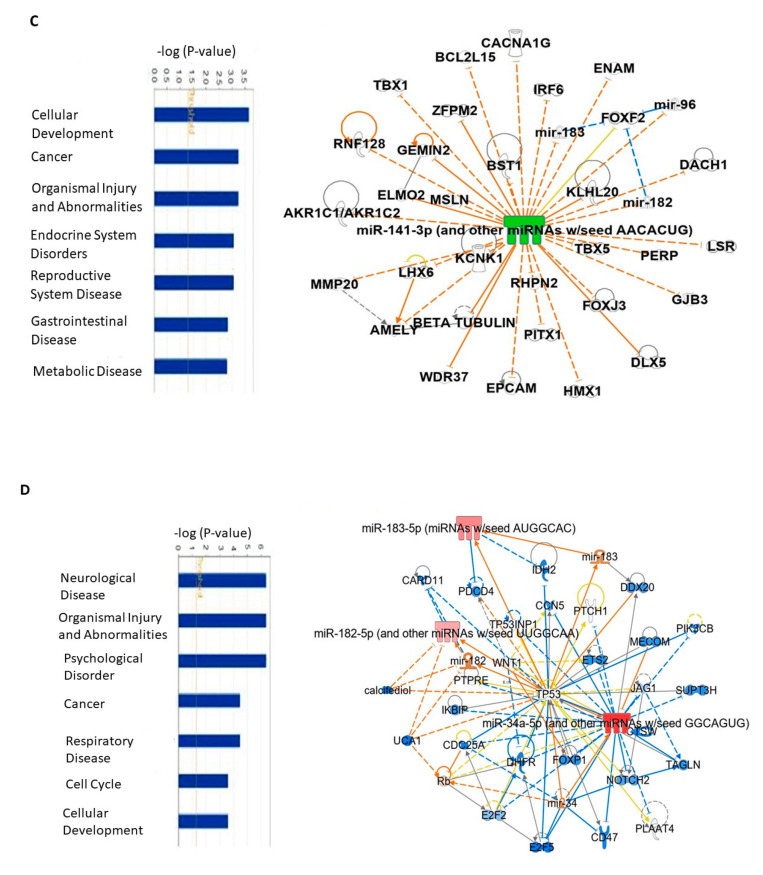
Exo-miRs-mechanistical networks resulting from IPA analysis. (**A**) IPA enriched network at baseline of exo-miRs of patients with pCR versus non-pCR; (**B**) IPA network at baseline of exo-miRs of patients with TNBC who achieved pCR versus those who did not; (**C**) IPA network after the first NACT dose of exo-miRs of patients with pCR versus baseline; (**D**) IPA network after the first NACT dose of exo-miRs of patients with non-pCR versus baseline.

The analysis of exo-miRs downstream effects using IPA showed multiple linked functions and diseases based on the published data. This analysis revealed enrichment in several categories associated with cancer, organismal injury, inflammation, connective tissue disorders, metabolic diseases, and cell cycle (Figure 7). Notably, it was revealed that the upregulated mir-30 at baseline in the group of patients with pCR was associated with downregulation of genes related to oncogenic functions, such as SYPL1, SLC38A1, ADAMTS-12, ADAMTS-14, ADAMTS-15, while the upregulated mir-127 at baseline in TNBC patients with pCR was associated with activation of estrogen receptor gamma (ESRRG), and inhibition of BCL6, NR0B2. After the initial NACT, the downregulated mir-141 was associated with positive regulation of several suggested tumor suppressors, such as IRF6, BCL2, KLHL20. The higher expression of mir-34 and mir-183 correlated with activation of TP53, and inhibition of NOTCH2, E2F3, CTSW, FOXP1, while the downregulation of mir-182 has the opposite effects.

**Figure 8:**
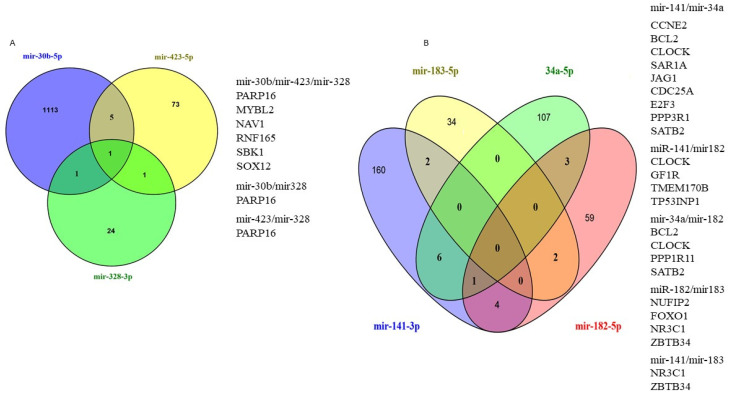
Venn diagrams showing the number of overlap target genes of exo-miRs at baseline (**A**) and after the first NACT dose (**B**) of breast cancer patients.

We have used TargetScan, miRDB, and mirDIP to predict the potential targets of exo-miRs and encountered large numbers of potential target genes of each of the selected miRNAs. The top putative overlapping in at least two of the databases [42] exo-miR target genes are shown in the Venn diagrams in Figure 8.

The target genes of exo-miRs at baseline and after the initial NACT included genes that have previously been associated with cancer. The top putative targets of the baseline exo-miRs included MYBL2, NAV1, SBK1, SOX12, PARP16, and RNF165. MYBL2 is a transcription factor and a physiological regulator of cell cycle progression, cell survival, and cell differentiation [43]. Overexpression of MYBL2 correlates with poor prognosis in numerous cancers [43], including breast cancer [44,45,46]. Recent studies have shown that MYBL2 was negatively regulated by mir-30b-5p in medulloblastoma [47]. NAV1 was reported to be significantly hypomethylated in ER+/PR+ breast cancers [48]. The top target genes of exo-miRs after the first dose of NACT included CCNE2, BCL2, CLOCK, SAR1, JAG1, CDC25A, E2F3, PPP3R1, FOXO1, SATB2, NR3C1, GF1R, TP53INP1, ZBTB34. CCNE2 plays a role in cell cycle progression and is aberrantly expressed in human cancers [49]. Overexpression of CCNE2 has been associated with pathogenesis [50], endocrine resistance [51], metastasis, and reduced survival [52] in breast cancer. BCL2 is upregulated by estrogens [53] in breast cancer, and its elevated expression has been associated with poor prognosis in luminal A breast cancer [54]. Loss of CLOCK gene, a transcription factor important in the regulation of circadian rhythm, has been associated with tumor progression in breast cancer [55].

**Figure 9:**
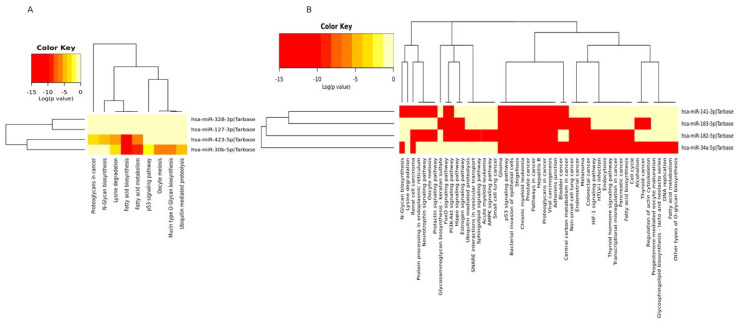
Heatmap of KEGG pathways enriched in the target genes of the four candidate miRNAs at baseline (**A**) and after the first NACT cycle (**B**) that were able to predict pCR. The isoforms of the seven-miRNA signature are involved in multiple cancer-related pathways (DIANA-mirpath computes log10 *p*-values).

KEGG pathway analysis at baseline showed that the potential target genes of mir-423 and mir30b shared enrichment in cell cycle, regulation of actin cytoskeleton, hippo signaling, adherens junction, pathways in cancer, while focal adhesion, proteoglycans in cancer, and carbon metabolism in cancer are associated with mir-127, and mir-423 (Figure 9A). After the first NACT cycle, the shared pathways between all four candidate miRNAs were pathways in cancer, prostate cancer, and viral carcinogenesis, while the shared pathways between mir-183, mir-34a, and mir-182 were endometrial cancer, colorectal cancer, glioma, melanoma, proteoglycans in cancer and hepatitis B (Figure 9B).

## 4. Discussion

In this study, we have used NGS to assess the global signature of exo-miRNAs in the plasma of breast cancer patients treated with NACT. We identified distinct exo-miRNA signatures that were capable to predict pCR before the start and after the first cycle of NACT. A four-miRNA signature before (hsa-mir-30b-5p, hsa-mir-328-3p, hsa-mir-423-5p, and hsa-mir-127-3p) and a four-miRNA signature after the first cycle of NACT (hsa-mir-141-3p, hsa-mir-34a-5p, hsa-mir-183-5p, and hsa-mir-182-5p) correlated with the therapeutic outcome in the breast cancer patients.

The results from this study fell in line with previous reports in which plasma was employed as a source of exo-miRNA biomarkers. The observed over-expression of mir-30b before the start of NACT in patients who achieved pCR correlates with the reported onco-suppressive functions of several members of mir-30s (miR-30a, miR-30b, miR-30c, miR-30d, and miR-30e) in breast cancer [56,57] and suggests for the first time that circulating mir-30 could potentially be a predictive biomarker of pCR prior to the start of NACT. The positive response to NACT in this study was also associated with the downregulation of miR-423-5p before the start of the treatment, a finding in agreement with previous studies showing that high tumor mir-423 correlated with poor prognosis [58,59,60]. A few studies have reported on mir-328 in breast cancer. Zeng et al. [61] showed that the expression of mir-328-3p negatively correlated with breast cancer metastasis. Significantly lower expression of mir-328-3p in breast cancer compared to normal tissues was found in TCGA database [62]. Thus, our novel finding of mir-328-3p downregulation prior to the start of NACT suggests its potential to serve as a predictive biomarker for NACT

This study showed that mir-127-3p was a strong predictor of the positive therapeutic response to NACT in TNBC. We have found a >4-fold higher expression of miR-127 prior to NACT in patients with TNBC who achieved pCR compared to those who did not. These findings correlate with previous reports showing that overexpression of mir-127 inhibits proliferation of breast cancer cell lines [63] of TNBC cell lines and suppressed metastasis. Several large neoadjuvant clinical trials on breast cancer showed that pCR varies among breast cancer molecular subtypes, and there was no correlation between the pCR rates and overall survival [7].

As we did not have access to tumor specimens, we accessed TCGA genomic data to examine the levels of the expression and the prognostic significance of exo-miRs in invasive breast cancer tissue. TCGA show that downregulation of the overexpressed in breast tumors mir-141, mir-182, and mir-183 is associated with better survival, which correlate with our finding of the lower expression of mir-141 after the initial NACT in the patients with pCR, and the higher mir-183 expression in patients with non-pCR. For example, overexpression of mir-141 significantly induced tumor growth, proliferation, and metastasis of breast cancer cell lines [64], and elevated expression of miR-141 was associated with shorter overall survival of breast cancer patients [65]. The aberrant expression of both mir-182-5p and mir-183-5p has been associated with tumor invasion and metastasis in various cancers [66,67,68], including upregulation in breast cancer [69,70]. Lower expression of mir-183 was reported in ER-positive compared to ER-negative breast tumors and higher in HER2-positive tumors compared to HER2-negative, suggesting the roles of miR-183 in different breast cancer cells are different [71]. The reported upregulation of mir-183 in TNBC versus the adjacent normal tissue [72] correlate with our findings of a negative correlation between the circulating exosomal and mir-183 and the positive response to the initial NACT in breast cancer patients. The downregulation of mir-182 after the first NACT cycle in patients with a negative response to NACT in our study correlates with the published [73] higher expression of mir-182 in breast cancer patients with three years free survival after NACT.

Breast cancer is a highly heterogeneous genetically and morphologically disease, which reflects in several staging systems, histopathologic classification, expression of hormones (ER, PR) and HER2 and/or BRCA mutation [74]. A recent review by Testa et al. [75] shows that at the molecular level, breast cancer is characterized “by high genomic instability, evidenced by somatic gene mutations, copy number alterations, and chromosome structural rearrangements,” which affect the survival outcome of breast cancer patients. Human miRNAs are frequently located at fragile sites and regions with a loss of heterozygosity or amplification associated with cancer [76]. For example, a study on breast cancer patient’s genomes showed a widespread loss of miR-3613-3p DNA fragment, located near the tumor suppressor genes RB1 and BRCA2 (13q13.1) [77]. Comparing the normal expression of mir-183, mir-141, mir-182, and mir-34a with their respective breast cancer subclass in TCGA, showed lower expression of these miRNAs in the luminal versus the other subclasses of breast cancer. TCGA also indicated differences in the expression patterns of several miRNAs based on the nodal metastasis status, including lower expression of mir-328, mir-423, mir-127, and mir-141 in patients with 3 positive lymph nodes versus the other nodal status (Supplemental Figure S2). Further, CNV gains of the onco-suppressors mir-30b and mir-127 were associated with better survival in comparison with the CNV deletions in breast cancer.

## 5. Conclusions

The results from this study suggest that significantly altered plasma exo-miRNAs prior to and after the first cycle of NACT may potentially serve as minimally invasive predictors of pCR in breast cancer. A four-exo-miRNA signature prior to (and four-exo-miRNA signature after the initial NACT dose correlated with the therapeutic response to NACT. Studies with a larger cohort of patients are needed to confirm the potential of these miRNAs as predictive biomarkers of NACT and their targets as liquid biopsy biomarkers for early prediction of the therapeutic response to NACT.

There are several limitations of this study. First, the study was performed on a small number of patients in each of the examined groups, which also restricted the analysis only to luminal A breast cancer and TNBC. Second, we did not include tumor samples and samples from normal breast tissue.

## Figures and Tables

**Table 1:** Patients characteristics.

	pCR	Non-pCR
Number of patients	6	14
Age (years), median (range)	52 (35–69)	52 (39–65)
Histology	IDC	IDC
Race		
White	4	12
Black	2	2
Tumor grade		
Grade I	1	1
Grade II	1	6
Grade III	4	7
Hormone status		
ER+/PR+/Her2-	3	11
ER-/PR-/Her2-	3	3

**Table 2:** Differentially expressed exo-miRNAs in plasma of breast cancer patients treated with NACT.

	Before NACT			After First Cycle of NACT		
	LogFC	*p*-Value	FDR	LogFC	*p*-Value	FDR
pCR						
hsa-miR-30b-5p	1.2025	0.0000	0.0039	−0.0499	0.8860	0.9996
hsa-miR-328-3p	−1.1040	0.0019	0.0360	−0.1081	0.7679	0.9996
hsa-miR-423-5p	−1.4271	0.0005	0.0127	−0.8390	0.0167	0.8232
hsa-miR-127-3p	4.5388	0.0000	0.0023	−0.0225	0.9514	0.9996
hsa-mir-141-3p	−0.1823	0.8631	0.9882	−2.5652	0.0000	0.0003
non-pCR						
hsa-miR-34a-5p	2.2219	0.0287	0.9883	3.0152	0.0000	0.0000
hsa-miR-182-5p	3.0821	0.0022	0.4906	1.2929	0.0000	0.0062
hsa-miR-183-5p	1.6985	0.0996	0.9883	1.7837	0.0000	0.0001

## Data Availability

The dataset generated and/or analyzed during the current study are available in the NCBI GEO database with the accession number GSE182951.

## Supplementary Materials

The following are available online at https://www.mdpi.com/article/10.3390/curroncol29020055/s1, Figure S1: Expression profiles of miRNAs with respect to breast cancer subclass. Figure S2: Expression profiles of miRNAs in relation to nodal metastasis status, Table S1: Review of the literature.
