# Supplementary material for: Circulating Exosomal microRNAs as Predictive Biomarkers of Neoadjuvant Chemotherapy Response in Breast Cancer
Source: Curr Oncol. 2022 Jan 28;29(2):613–30. doi: 10.3390/curroncol29020055 (PMC8870357; doi:10.3390/curroncol29020055)
Supplement: Supplementary file 1 [file curroncol-29-00055-s001.zip › curroncol-1512088-supplementary.pdf]

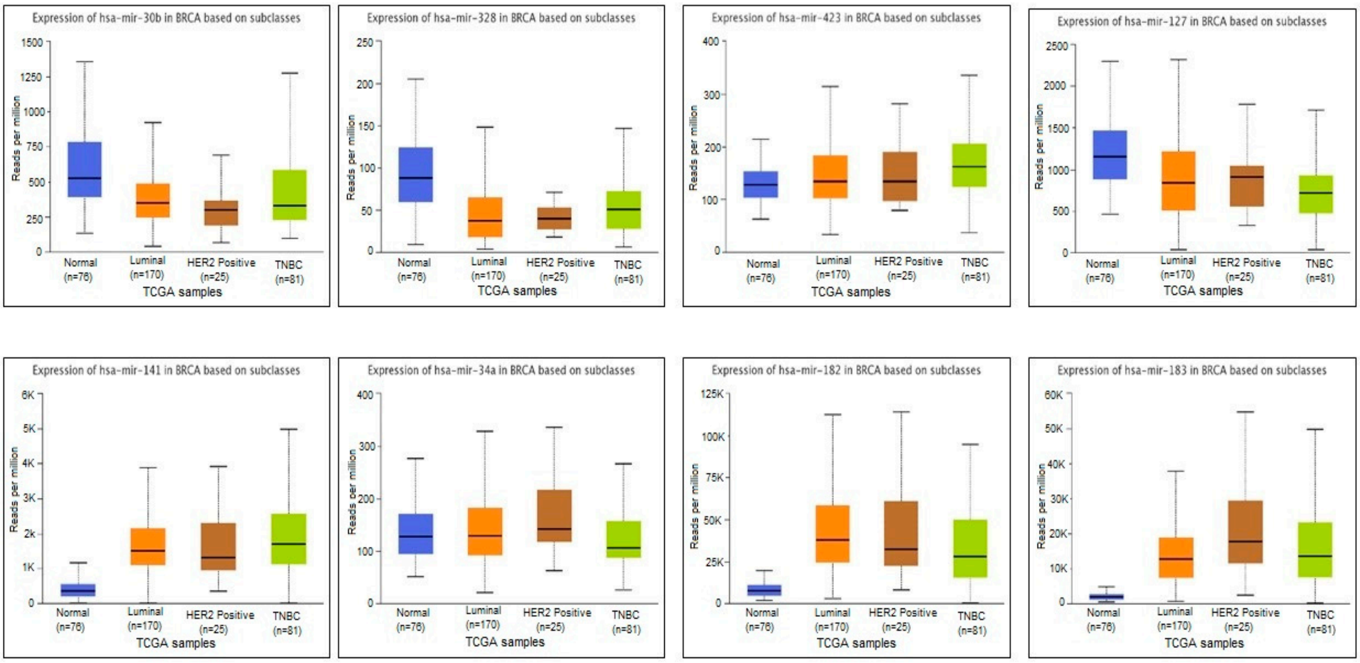

Figure S1. Expression profiles of miRNAs with respect to breast cancer subclass.

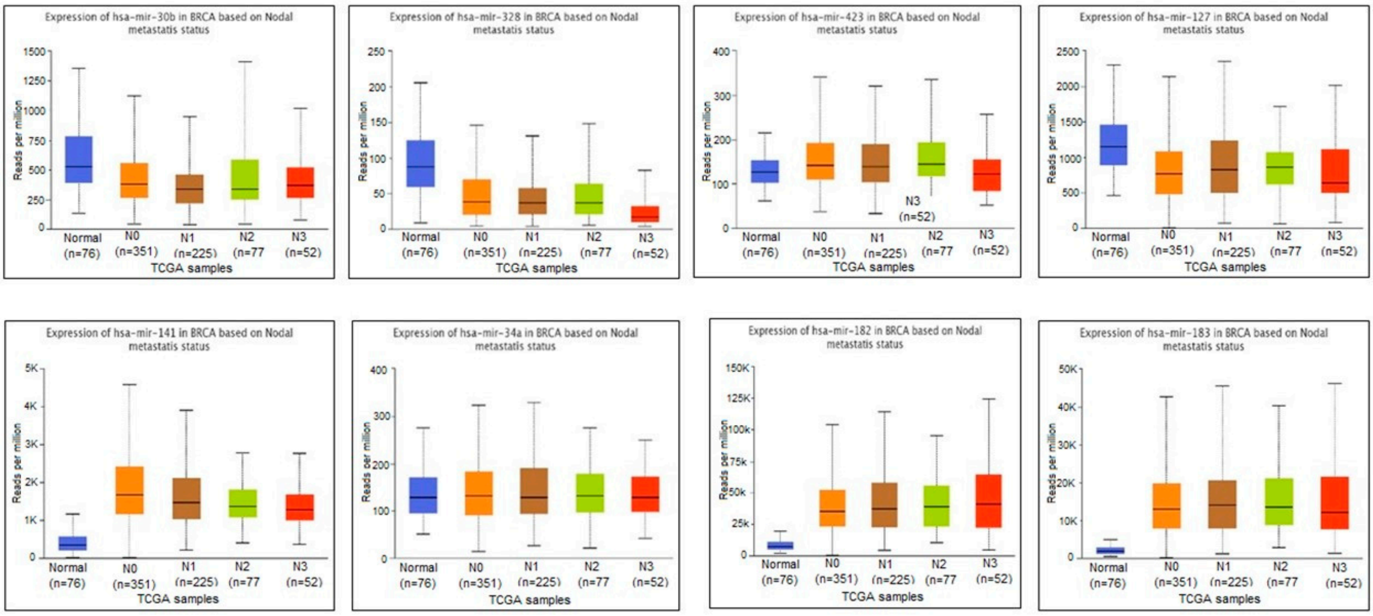

Figure S2. Expression profiles of miRNAs in relation to nodal metastasis status.

Table S1. Review of the literature. Publications in Pubmed on mir-30b-5p, mir-423-5p, mir-328-3p, mir-127, mir-141, mir-34a, mir-182 and mir-183 in breast cancer.

miRNA	Tumor tissue	Expression in breast cancer (BC)	PMID
mir-30b	Serum and urine extracellular vesicles from TNBC patients and TNBC cell lines	Downregulation in cells treated with epirubicin, gemcitabine and paclitaxel; downregulation in untreated TNBC versus healthy controls, no clear pattern in the expression into responders and non-responders	31789396
	Plasma exosomes	Downregulation associated with recurrence	30154547
	Serum	Downregulated in BC patients comparison with healthy controls	24866395
	BC cell lines	Upregulated (including its target CCNE2) in trastuzumab resistant BC calls.	28120942
	Primary tumors	Downregulation of mir-30 family members associated with poor relapse-free survival	30042152
	BC cell lines and tumor tissue	Upregulation promoted cell proliferation and invasion in TNBC cell lines, but inhibited apoptosis via targeting ASPP2 and AKT pathway	32420372
	Whole blood	Upregulated in patients with early BC in comparison with healthy controls	28359916
	Tumor tissue from luminal BC without metastasis	Higher in sensitive to endocrine treatment tumors in comparison with the resistant tumors	31572437
	Tumor tissue	Downregulated in tumors in comparison with the adjacent non-cancer tissue.	22524830
	Plasma	Age-related downregulation	25333486
	Plasma and cancer tissue versus adjacent normal tissue	Overexpression in advanced disease, both in plasma and tumor tissue versus controls	31888645
mir-423	Tumor tissue and normal tissue, BC cell lines	Upregulation enhanced viability, migration and angiogenesis of breast cancer cells	31213129
	BC cell lines, tumor tissue	Upregulated and correlate with poor prognosis	32606763
	BC cell lines	Upregulated in exosomes from chemoresistant to cisplatin TNBC cells	31424364
	BC tissue	Upregulation in surgical specimens versus pre-neoadjuvant biopsies	27746365
	BC cell lines, tumor tissue	Upregulated in chemoresistant cells, activate ZFP36	32158228
	Plasma, during and after doxorubicin NACT chemotherapy	Upregulation associated with chemotherapy-related cardiotoxicity	29378531
	PubMed, EMBASE and Web of Science databases	Polymorphisms associated with increased or decreased risk of BC	28415619
	Plasma	Age-related downregulation	25333486

mir-328	BC cell line, tumor and adjacent tissue	Downregulated in BC tissue	29620238
	Tumor and adjacent tissue, BC cell lines	Downregulated in BC tissue, regulate GRM4	31492116
	BC cell lines	Downregulation associated with ABCG2 overexpression in mitoxantrone-resistant BC cells	19270061
mir-127	TCGA BRCA (GEO: GSE19536, GSE38167, GSE59829)	Downregulation in TNBC tumors compared to normal tissue; decreased in lymph node metastasis in TNBC	31694904
	BC tissue and tumor-surrounding normal tissue	Downregulation correlated with lymph node metastasis and advanced clinical stage. Upregulation inhibited growth, enhanced apoptosis and reduced invasion by targeting BCL-6	25477702
	Breast cancer cell lines TNBC	miR-127 prodrug suppressed growth and metastasis in TNBC, increased disease free survival	31694904
	BC cell lines, BC tissues and tumor-surrounding normal tissue	Downregulation was associated with upregulation of BCL-6	24282530
mir-141	Needle-aspiration tumor samples	Upregulated in tumors before taxene-anthracycline NACT in patients with non-pCR. Elevated mir-141 predict poor clinical response to taxene-anthracycline neoadjuvant chemotherapy	26078798
	Serum	Upregulation associated with shorter brain metastasis survival	27075851
	Plasma	Upregulation associated with metastasis, regulated by FOXP3-KAT2B axis	28637482
	BC cells	Upregulation conferred resistance in docetaxel-sensitive cells, correlated with EIF4E expression changes	25813250
	Whole blood	Upregulation in the blood of patients with stage I-III, lymph node metastasis, and HER2 negative tumors	25885099
	BC cell lines	Downregulated in trastuzumab –resistant cells by affecting expression of CDK8	31087707
	BC cell lines	Downregulated in trastuzumab –resistant cells by upregulating ERBB4	30746756
	Tumor tissue	Upregulated in doxorubicin-resistant BC tissue	25451164
	BC cell lines	Upregulated in docetaxel-resistant cells	21399894
	Plasma	Upregulated in plasma of circulating tumor cells (CTC)-positive patients with metastatic BC compared to CTC-negative patients	22952344

mir-34a	Plasma and tumor tissue	Upregulated in plasma and tumor tissue after NACT in patients with pCR, especially increased after anthracycline-based chemotherapy	25078559
	Blood	Downregulated after two cycles of NACT in patient insensitive to chemotherapynot-sensitive to	30099860
	Metastasizing BC cell lines and tumor tissue from mice and patients	Downregulated in metastatic BC cells; overexpression inhibits migration and invasion in vitro, metastasis suppressor in vivo	23001043
	Plasma from TNBC and healthy controls	Downregulated compared to healthy controls, low expression associated with worse overall survival	28540980
	BC cell lines	Upregulated in docetaxel-resistant cells	21399894
	BC cell lines and tumor tissue	Downregulated in all BC cells; in tumors mir-. inversely correlated with ErbB2 levels,	27813227
	BC cell lines and tumor tissue	Downregulated in multidrug-resistant BC cell line; patients with low expression had poorer overall survival	29290947
	Serum from BC patients and healthy volunteers	Downregulated in BC patients in comparison with the controls; correlated with tumor stage and hormone receptor status	29854296
mir-182	DCIS (non-invasive/pre-invasive BC) (ER/PR-positive/HER2 negative) and paired normal epithelium from reduction mammoplasty	Overexpressed in DCIS compared to normal epithelium	21375733
	Breast and prostate cancer cells	Mir-182 can be transferred between cells, preferentially packaged in exosomes	27446418
	Breast cancer cell lines	Overexpressed in MCF-7 breast cancer cells, associated with downregulation of FOXO1	19574223
	TNBC tumor samples, BC cell lines and animal experiments	Upregulated in TNBC tissue and cell lines; promoted tumor metastasis in mice; FOXF2 identified as direct target	29085483
	Tumor tissue and BC cell lines	Upregulated in BC tissue associated with poor survival; abrogation of mir-182 inhibited cell proliferation and invasion in BC cells	30666836
	Trastuzumab-resistant BC cells	Downregulation in trastuzumab BC cells, direct target of mir_182 was MET	29925897
	BC cells, murine xenograft model	Upregulated in BC cells and tissue, associated with poor survival; depletion of mir-182 was associated with up-regulation of PTEN in murine model	30666836

Mir-183	BC cell lines	Upregulation associated with downregulation of RB1 and enhanced proliferation and migration of BC cells	28693273
	DCIS (non-invasive/pre-invasive BC) (ER/PR-positive/HER2 negative) and normal epithelium from reduction mammoplasty	Overexpressed in DCIS compared to normal epithelium	21375733
	BC tissue, BC cells	Upregulated in BC tissue and negatively regulated FHL1 in BC cells	32908787
	BC tissue and BC cells	Upregulation in BC tissue versus the adjacent normal tissue, enhanced BC cells proliferation and migration, and inhibited apoptosis; PDCD4 as a putative target	27476679
	TNBC and surrounding tissue, BC cells	Upregulated in TNBC tissue	24788655
	Breast and prostate cancer cells	Mir-183 can be transferred between cells, preferentially packaged in exosomes	27446418
	Retrospective case study on BC	Upregulation was associated with poor prognosis	25277099
